# A longitudinal analysis on alcohol consumption in patients with cancer undergoing psycho-oncological treatment

**DOI:** 10.1038/s41598-025-04849-w

**Published:** 2025-06-20

**Authors:** Paulina Kiefer, Lisa Lebherz, Janina Freitag, Holger Schulz, Christiane Bleich, Carsten Bokemeyer, Frederike Bokemeyer

**Affiliations:** 1 Department of Medical Psychology, University Medical Center Hamburg Eppendorf, Martinistraße 52, 20246 Hamburg, Germany; 2 Center for Oncology, II. Medical Clinic and Polyclinic, University Medical Center Hamburg Eppendorf, Martinistraße 52, 20246 Hamburg, Germany; 3 Department of Psychosomatic Medicine and Psychotherapy, University Medical Center Freiburg, Hauptstraße 8, 79104 Freiburg, Germany

**Keywords:** Cancer survivorship, Alcohol consumption, Psycho-oncology, Longitudinal analysis, Lifestyle, Health behaviors, Tertiary prevention, Supportive care, Oncology, Risk factors

## Abstract

The negative impact of alcohol consumption on cancer development and progression is well-established in oncologic research, yet it receives surprisingly little attention from patients with cancer, the public, and even oncology professionals. A cancer diagnosis can lead to significant psychological distress, including high levels of depression and anxiety. For patients with cancer experiencing high levels of psychological burden, psycho-oncological care is available to help manage these symptoms and the overall impact of their condition. Alcohol consumption can serve as a coping mechanism for psychological stress. However, there is limited knowledge about the alcohol consumption patterns among this particularly vulnerable group of patients with cancer, as well as the patient- and disease-related factors associated with drinking. Patients with cancer are particularly susceptible to the harmful effects of alcohol. The aim of this study is to investigate the prevalence of potentially risky alcohol consumption among patients with cancer receiving psycho-oncological care over a six-month period and to identify sociodemographic, health-related, and psychosocial factors that may predict alcohol consumption after a cancer diagnosis. We conducted a secondary analysis using data from 300 patients with cancer (72 % female, mean age 52.74 years) treated at the outpatient clinic of the University Medical Center Hamburg-Eppendorf in Germany. Between 2013 and 2021 demographic, medical, and psychosocial information was collected using self-report questionnaires. A generalized longitudinal linear mixed model was used to determine the prevalence of risky and potentially harmful drinking behavior (AUDIT-C ≥ 2 for women and ≥ 3 for men) among patients with cancer as well as to identify patient characteristics associated with alcohol consumption. The results show that approximately 70% of the patients continued drinking after their cancer diagnosis, despite the known detrimental effects of alcohol on prognosis. At both time points, around 40 to 50% of female and male patients reported potentially harmful drinking behaviors (T0 (beginning of psychosocial treatment): 49.1% of female, 38.1% of male patients; T1 (6 months later): 41.2% of female and 42.9% of male patients). A higher number of comorbidities (OR = 0.707; 95% CI: 0.567–0.883), older age (OR = 0.983, 95% CI: 0.967–0.999, and higher levels of depressive symptoms (OR = 0.952, 95% CI: 0.907–0.998) were significantly associated with lower odds of risky alcohol consumption over the six-month period. In contrast, higher anxiety levels (OR = 1.075, 95% CI: 1.021–1.132) were associated with an increased likelihood of risky drinking. The significant proportion of patients with cancer consuming alcohol at levels that may worsen their cancer prognosis highlights the need for improved patient education and guidelines. The results can help identify high-risk patients who require close monitoring of their drinking behaviors during their survival period, and inform the implementation of better alcohol control measures in cancer care. By understanding alcohol consumption patterns and associated factors, we aim to promote healthier behaviors and improve treatment outcomes for patients with cancer in psycho-oncological care.

**Supplementary Information:**

The online version contains supplementary material available at 10.1038/s41598-025-04849-w.

## Introduction

Approximately 5% of all cancer diagnoses and 6% of cancer deaths worldwide can be attributed to the use of alcohol^1–4^. In Germany alone, more than 20,000 cancer cases are caused by alcohol consumption each year^5^.

Alcohol is a known risk factor for several types of cancer, including cancers of the oral cavity, pharynx, larynx, esophagus, liver, colon, and female breast^2,6–8^. Its’ carcinogenic effects are numerous and well-studied, with a major factor being the irreversible DNA damage it causes to cells^2,9^.

For a number of cancer types, alcohol consumption is associated with an increased risk of developing a second primary cancer, recurrence, and decreased survival, even when controlling for tumor stage^10–15^. Also, the risk of death is higher for patients who continue to drink after a cancer diagnosis, especially for those suffering from cancers strongly associated with alcohol consumption^13–15^.

However, the importance of alcohol consumption as a factor contributing to the overall cancer burden is often underestimated^16^, and awareness of the harmful effects of alcohol on both the development and the progression of cancer is surprisingly low among patients, the general population, and even oncology professionals^17,18^.

Concurrently, lifestyle and health behaviors are increasingly recognized as important strategies for improving survival, reducing disease burden, and slowing disease progression. They include diet, physical activity, smoking and also alcohol consumption^19^. The clear evidence that alcohol, even at low levels of consumption, contributes to both, the development and progression of several types of cancer^20–22^ and can negatively impact health outcomes and cancer mortality^23,24^, together with the high prevalence of alcohol consumption among cancer survivors, highlights the need for further research into patients’ drinking patterns and the development of strategies to reduce alcohol consumption in this vulnerable group.

Particular attention should be paid to the post-diagnosis period, as cancer survivors may be motivated to adopt healthy behaviors after their diagnosis^12,25,26^, promoting the idea of a ‘teachable moment’^12^. Support services, such as psycho-oncology treatment, may also provide a good framework for managing risk behaviors and initiating behavioral change^27^.

Despite this, there is a lack of appropriate guidelines on alcohol consumption for patients with cancer and there is still confusion about what level of consumption is associated with a worsening of cancer. The American Institute for Cancer Research (AICR) and the World Cancer Research Fund (WCRF) consider there to be no safe level of alcohol consumption in terms of cancer risk (i.e. progression and recurrence)^2,28^ and recommend total abstinence for patients, as there is no evidence for a specific threshold of risk-free or even beneficial drinking levels^29^. Likewise, in August 2024, the German Society for Nutritional Medicine published new data emphasizing that no level of alcohol consumption can be considered safe and recommending complete abstinence from alcohol even for the general population^30^. However, studies have found a high prevalence of current drinkers and also excessive drinking behaviour among patients with cancer^31,32^, with no significant difference from non-cancer controls^33,34^. A large number of patients (up to 60%) maintain their alcohol consumption after receiving a cancer diagnosis^35–37^. Especially for heavy users, a cancer diagnosis seems to only have little impact^38^. This contrast underscores the urgent need for further efforts in patient education and tertiary prevention.

Although the percentage of non-drinkers increased over the course of survival^39^ and reductions in consumption were reported in some studies^33,40^, improvements were not maintained over time^26,33,34^. Previous research on alcohol consumption in patients with cancer has identified a number of factors that influence drinking patterns:

### Time since diagnosis

Several studies have found no association between hazardous or current drinking and the time since diagnosis^15,31,41^. If a decrease in alcohol consumption was observed in the first 2–4 years after diagnosis, drinking levels and the number of current drinkers increased again later on^26,38,42^.

### Gender

Across the current literature on both patients with cancer and non-cancer controls, men show a consistently higher prevalence of alcohol use, with higher odds of current drinking, hazardous drinking and heavy drinking^43,44^.

Women drink at significantly lower levels and are more likely to quit drinking^43^. Interestingly, some researchers have found that the continuation of drinking after a cancer diagnosis is not influenced by gender^35,42^. Then again, some research indicates that women may be more likely to reduce or abstain from alcohol consumption after a cancer diagnosis compared to men. They will be more likely to refrain from excessive drinking in the post-diagnosis period^45^. Factors such as concerns about overall health, treatment side effects, and a desire to adopt healthier lifestyles may contribute to these changes in drinking behavior.

### Age

Older patients with cancer (65 + years) were less likely to be current, high-risk, or heavy drinkers^34,36,37,46^. On the other hand, the prevalence of high-frequency drinking, such as daily consumption, tended to increase with age and was consistently higher in older patients^16^.

### Type of cancer

There is a higher proportion of high-risk drinkers among patients with alcohol-related cancers. However, in previous studies, these patients also had a lower prevalence of current drinking after diagnosis and were more likely to quit drinking after cancer diagnosis^38,47^.

### Cancer treatment

Patients who underwent chemotherapy, radiation or a combination of the two were significantly less likely to engage in hazardous alcohol consumption in the early post-diagnosis period than those who underwent surgery alone^42^. This is likely due to the prolonged and severe side effects of systemic therapies such as radio- or chemotherapy.

### Number of comorbidities

A number of studies found no association between alcohol consumption and the number or severity of comorbidities^15,42^. However, Tollosa et al. (2020) reported that a higher number of comorbidities was significantly associated with poorer health behaviors, including higher alcohol consumption^26^.

### Self-perceived health/ quality of life

Some studies have reported that the highest prevalence of hazardous drinking was found among patients with cancer with good self-perceived health^15,41^.

Researchers have also found a positive correlation between alcohol use and quality of life in patients with cancer. Then again, this was particularly associated with good nutrition and oral function^35^. It is possible that patients whose health status allowed them to maintain their daily routines and participate in social activities may perceive themselves less at risk for the harmful impacts of alcohol consumption after a cancer diagnosis and will be more likely to drink alcohol. On the other hand, problem drinkers had the worst quality of life and highest prevalence of depressive symptoms^35^.

### Educational status and income

Previous researchers have reported that a lower educational status is associated with poorer health behaviors in patients with cancer^26^. Those with less education were less likely to meet the recommended guidelines for alcohol consumption^47^. Others found the highest prevalence of hazardous drinking in women with the highest educational attainment^48^. Additionally, patients with higher family income had significantly higher alcohol consumption^31,36,49^.

### Children and partnership

In studies of non-cancer populations, parents had lower rates of hazardous drinking than those without children. Drinking was further reduced with a higher number of children^49^. While some studies found no association between marital status (partnership) and alcohol use among patients with cancer^15,40^, others reported that married individuals were more likely to be non-drinkers and less likely to exceed moderate drinking or engage in binge drinking^31^.

### Depression and anxiety

Patients with cancer generally show significantly increased prevalence of both depressive and anxiety symptoms^50^.

In other studies, alcohol use and abuse were significantly associated with depressive symptoms in patients^35^. Among older male patients with advanced cancer, the presence of depressive symptoms was found to be a significant predictor of higher alcohol consumption^51^. A positive association between alcohol consumption and anxiety has also been reported in other research^52,53^.

It is relevant to take a closer look at alcohol consumption in patients with cancer receiving psycho-oncologic treatment, as this group of patients shows high levels of depressive symptoms and high psychological distress, resulting from complex individual, social, disease-related and treatment-related factors^50,54^.

#### Patients with cancer in psychological care

The population of patients with cancer has been extensively studied, but little attention has been paid to those who are particularly burdened, such as those already receiving psycho-oncology care. It is well documented that a cancer diagnosis often results in significant distress, including a high prevalence of psychological symptoms such as depression and physical symptoms such as fatigue^50,54^. Increased levels of distress, depression and anxiety are known to be associated with increased alcohol consumption and excessive drinking in the general population, where alcohol is often used as a coping mechanism^55,56^.

Patterns of alcohol use and the potential role of alcohol as a coping strategy among patients with cancer receiving psycho-oncological treatment are not well documented. These patients are likely to experience high levels of psychological distress and disease burden^57^. Psycho-oncology treatment offers self-reflection, cognitive restructuring, and psychoeducation to promote healthier lifestyles. By reducing symptoms of depression and anxiety and helping patients develop healthier coping mechanisms, psycho-oncology care may also positively impact alcohol use in this group of patients with cancer^50,54^. In addition, patients receiving psycho-oncology treatment may be more motivated to engage in healthy behaviors that could positively influence their disease course^57,58^.

#### Study objectives

The Cancer Prevention Committee of the American Society of Clinical Oncology recently emphasized the importance of evaluating and accurately measuring the impact of alcohol on cancer treatments and assessing patients’ drinking habits^16,59^. To date, research on the health and drinking behavior of patients with cancer has been inconclusive, with a particular lack of information on cancer patients undergoing psycho-oncology treatment.

Focusing on this special patient population, our goal was to identify patient characteristics associated with alcohol consumption and to determine if drinking behavior changes over the course of treatment.

## Materials and methods

### Study design

A secondary analysis of longitudinal data (at least baseline and 6-month follow-up) was performed to identify sociodemographic, medical, and psychosocial variables associated with alcohol consumption patterns of psycho-oncology patients.

### Participants and procedure

The analysis used routine data from patients with cancer who visited the psycho-oncological outpatient clinic of the Department of Medical Psychology at the University Medical Center Eppendorf in Hamburg, Germany, between 2013 and 2021. In this outpatient clinic, patients receive individual support for their psycho-oncological symptoms and are routinely given self-assessment questionnaires that collect information on sociodemographic, medical, and psychosocial aspects. The questionnaires are part of the clinical care and have the purpose to develop a targeted treatment plan and tailor psycho-oncology care to the individual needs of patients. Therefore they collect voluntary information about patients physical, mental, and psychosocial well-being and also include as a brief screening instrument for alcohol use. A first questionnaire is handed prior to the first visit of the outpatient clinic and a follow-up questionnaire is used six months later to document any changes in patients’ health status, treatment, and well-being.

**Fig. 1:**
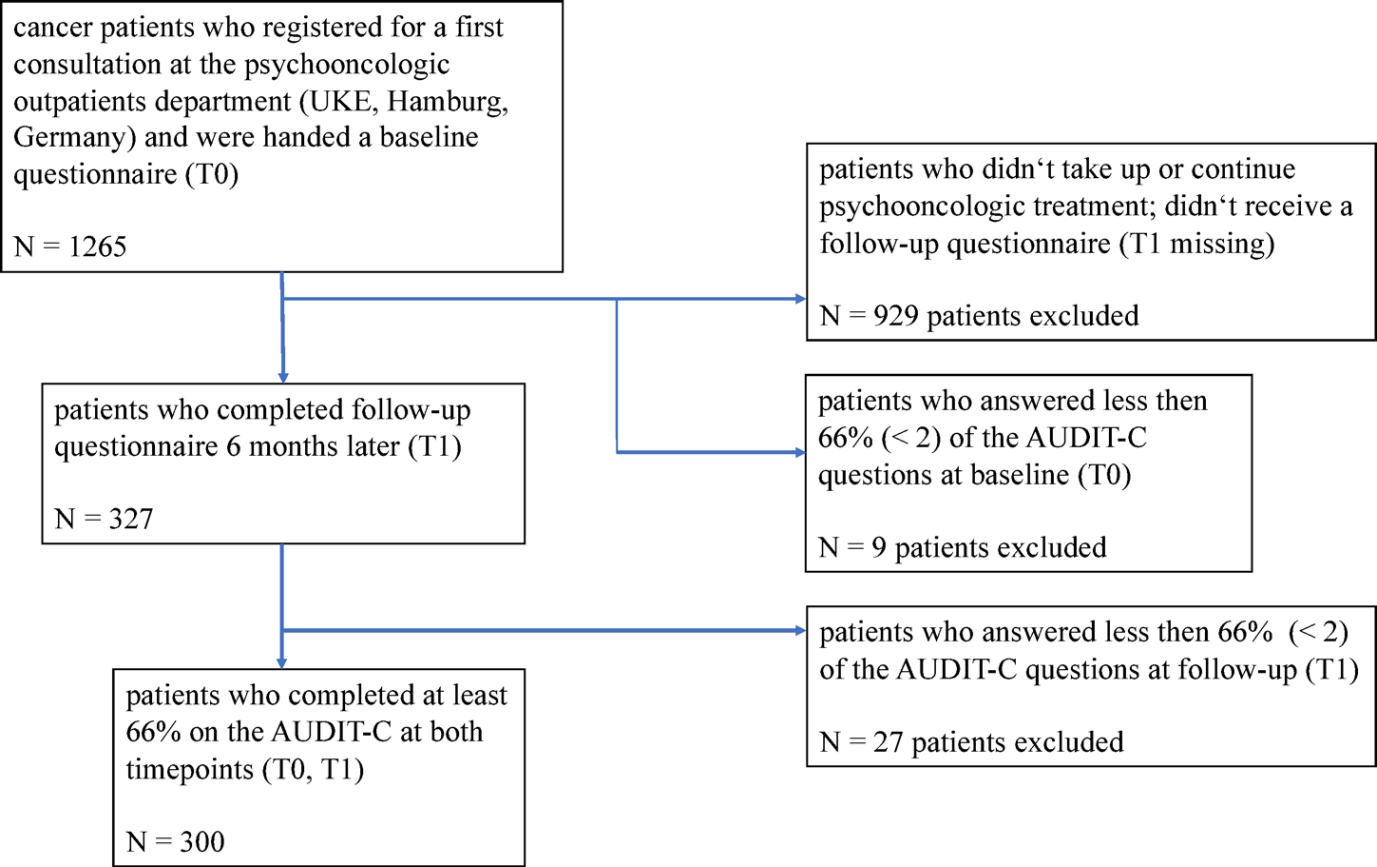
Flowchart of study population and patient recruitment.

Between 2013 and 2021, 1265 patients with cancer and their family members received a first questionnaire. 327 of the patients completed both the baseline and follow-up questionnaires. As participants with a response rate below 66% were excluded from inferential statistics, this lead to a total of 300 participants that were included in our analysis. An overview about the study population and recruitment is given in Fig. 1.

Especially in the beginning, difficulties in delivering the second questionnaire occurred. Reasons for dropout during the follow-up process included high physical and psychological burden of the disease, lack of interest, or death.

As this analysis was conducted retrospectively using data collected from the outpatient clinic, this study qualifies as a secondary analysis. Inclusion criteria for the analysis were a cancer diagnosis of any type and completion of at least two of the three questions on alcohol consumption. The minimum age of the participating patients was 18 years. During the observation period, patients had an average of 6.8 contacts with psycho-oncology caregivers. The most common reasons for psycho-oncological counselling included coping with the disease, uncertainty about the prognosis, and anxiety (particularly regarding progression and fear of death)^60^.

### Ethical considerations

Patients gave written informed consent for data to be used anonymously for research purposes.

The study was conducted in accordance with the tenets of the Declaration of Helsinki. In addition, the study was approved by the Ethics Committee of the LPEK (Local Psychological Ethics Committee at the Center for Psychosocial Medicine Hamburg, Germany) (tracking number: LPEK- 0284).

## Measures

### Assessment of demographic information

Demographic and health information was categorized as follows: age (continuous), gender (male, female), partnership status (do you live in a partnership, alone or other), highest level of education or training (university or college degree; technical college or apprenticeship; no further education), and presence of children (yes/ no). Information on education level and having children was only collected in the baseline questionnaire. Note that in these earlier questionnaires, the gender option was only male or female. The updated version now also includes the option ‘diverse’.

An overview of the demographic, medical and psychosocial information collected at baseline is given in Table 1.

**Table 1:** Baseline assessment of covariates.

Covariate	Scale	Levels
Demographic information*		
Gender	Categorical	Male Female
Age	Continuous	Age range (0 – highest)
Partnership	Categorical	In a partnership Single Other
Educational level	Categorical	University or college degree Technical college/ apprenticeship No further education
Children (parenthood)	Categorical	Yes/ no
Medical information*		
Time since diagnosis	Continuous	Year range
Number of comorbidities	Continuous	Number range
Use of psychopharmaceuticals	Categorical	Yes/ no
Type of cancer treatment	Categorical	Radiation therapy, chemotherapy, or stem cell therapy (also combined) Surgery Pain or antihormonal therapy
Alcohol-related cancer type	Categorical	Alcohol-related Not specifically alcohol-related
Psychosocial variables		
Distress thermometer (DT) psychosocial distress over the past week (83–85)	Continuous	Scale range 0–10 (0 = no distress, 10 = extreme distress) ≥ 5 indicates relevant level of distress, need for further support for patients (83,86)
GAD-7 anxiety symptoms and assessment of symptom severity (87)	Continuous	Scale range 0–21 Categories for symptom severity (87): Minimal (0–4 points), Mild (5–9 points), Moderate (10–14 points), Severe (15–21 points) ≥ 7: relevant levels of anxiety (88)
PHQ-9 depressive symptoms and symptom severity (89–91)	Continuous	Scale range 0–27 Categories for symptom severity: Minimal (0–4), Mild (5–9), Moderate (10–14), Severe (15–27) ≥ 10: relevant depressive symptoms (89)
SF-8* health-related quality of life including physical and mental aspects, e.g., symptoms, activities of daily living, mental well-being of patients (92,93)	Continuous	Scale range 0-100; Mean (SD): 50 (10); representing a standard mean for health and functioning (93–95)
EORTC29/30* cancer-related overall health (29) and quality of life (30) during the past week from EORTC QLQ-C30 (Quality of Life of Cancer Patients) Core Questionnaire (96)	Continuous	Scale range 1–7 1 = ‘very poor’; 7 = ‘excellent’ overall health and quality of life (96)
AUDIT-C alcohol consumption (61)	Categorical	Cut-off risky consumption for women: AUDIT-C ≥ 2 (‘Group 2’) vs. scores below (‘Group 1’), Cut-off risky consumption for men: AUDIT-C ≥ 3 (‘Group 2’) vs. scores below (‘Group 1’)

* Items only included with baseline information and hold constant within the analysis.

### Assessment of medical information

Medical information on patients’ disease and treatment status included time since cancer diagnosis, number of comorbidities (all continuous), use of psychopharmaceuticals, type of cancer, and type(s) of treatment (categorical). For analysis, the sample was further divided into two groups according to evidence in the literature of an association between alcohol consumption and cancer development. The group with alcohol-related cancers included head and neck cancer, female breast and gastrointestinal cancers^2^.

Regarding cancer treatment, the following categories were defined: received radiation therapy, chemotherapy, or stem cell therapy (also combined); underwent surgery; or received pain or antihormonal therapy. For radiation therapy and chemotherapy, patients were included if they were either currently receiving one of these therapies, had received one within the past, or selected “applicable” for these options in the questionnaire.

### Assessment of psychosocial variables

Table 1 gives an overview of the psychosocial assessment variables. For the DT, GAD-7, PHQ-9, SF-8, and EORTC29 and 30 individual sum scores were included in the analysis.

#### Alcohol consumption

Information on the patients’ drinking behavior was collected using the AUDIT-C questionnaire, an abbreviated version of the Alcohol Use Disorder Identification Test (AUDIT). This screening tool has been validated to detect risky drinking, harmful drinking and alcohol use disorders^61,62^. This short questionnaire consists of three questions, inquiring about the quantity and frequency of alcohol consumption, as well as the occurrence of binge drinking in the past 12 months.

It has been validated as an effective screening tool to identify risky drinking^15,63,64^. Total scores can range from 0 to 12, with 0 indicating no alcohol consumption and 12 indicating high levels of consumption. Patients with an AUDIT-C score of 0 have not consumed any alcohol in the past 12 months and can be considered abstainers. A score of 1 indicates occasional use of approximately 1 to 2 glasses of alcohol once a month or less. Scores of 2 to 4 represent light to moderate drinking levels, and scores above 5 indicate heavy drinking. A score of 3 might reflect drinking 1–2 drinks per week, or larger amounts consumed less frequently. A score of 4 may also reflect light but regular drinking, or occasional heavy drinking. Previous research has discussed different cut-off points for risky consumption, varying from 3 to 5 for both men and women^32,65,66^.

However, applying these recommendations to patients with cancer requires reevaluation. Based on the most recent data emphasizing that no level of alcohol consumption can be considered ‘safe’, the potential health risks of even low levels of consumption are being highlighted^67,68^. Currently, there is a lack of robust data on how specific levels of alcohol consumption affect cancer prognosis in general, including possible gender differences within this patient population. For the general population, differences in alcohol tolerance between women and men are reflected in the cut-off scores for this screening instrument, and these differences are likely also relevant for patients with cancer^69^.

Until further research has examined these associations in more detail, the consumption of alcohol must be considered potentially harmful for patients with cancer.

For this analysis we lowered the standard threshold for by one point and applied gender-related differences, defining risky drinking as a cut-off score of 2 and higher for women and 3 and higher for men.

The sample was divided into two groups accordingly for the analysis: ‘Group 2’ included all patients with cancer who reported risky and potentially harmful drinking behavior, such as all regular moderate and regular consumption as well as heavy and binge drinking. ‘Group 1’ included all patients who did not meet the criteria for risky drinking behavior.

### Statistical analysis

Data management and analyses were performed using SPSS, version 30.

Patients were excluded from inferential statistics if their indicidual response rate was below 66% at a given time point. To handle missing data, expectation maximization algorithms (EM) were applied prior to conducting descriptive statistics^70^. Misssing values in psychosocial and clinical self-report measures were imputed using EM, provided that the patient’s reponse rate was at least 66.6%. This threshold was defined a priori. If the response rate fell below this level, no imputation was performed.

Following imputation, descriptive statistics were used to summarize the sociodemographic and medical characteristics of the patients with cancer at baseline. Means with standard deviations (SD) and ranges were used for continuous variables. For categorical variables, counts and percentages were calculated.

Patients’ mean alcohol consumption and other psychosocial measures were described at both time points to report the 6-month trends. The primary endpoint of the analysis, alcohol use, was patients’ total AUDIT-C score. A binomial distribution was used and categories were recoded as follows: *‘group 1*’ (AUDIT-C score of 0 or 1 for women and 0, 1 or 2 for men), and ‘*group 2’* (AUDIT-C scores of 2 and higher for women and 3 and higher for men). The dependent variable in the model calculation was *‘group 2’*, representing risky or potentially harmful drinking patterns in patients with cancer.

A generalized longitudinal linear mixed model (GENLINMIXED command in SPSS) including patients’ demographic, clinical, and psychosocial characteristics was used to identify factors associated with consumption patterns (dependent variable ‘*group 2*’). The mixed model procedure allows for both fixed and random effects (IBM SPSS Statistics, 2022) and produces robust estimators for the coefficients of the fixed effects.

A normal distribution of the residuals is not required^71^. Model 1 included standard fixed effects. Time point, age, time since diagnosis, number of comorbidities, EORTC29, EORTC30, SF-8, Distress Thermometer, GAD-7, PHQ-9 and number of psychotropics used were included as continuous variables. Gender, partnership status, children (parenthood), educational status, cancer type (alcohol-related yes/no) and cancer treatment modality were categorical. The multilevel analysis did not impose any restrictions on the number of levels per variable. To ensure focus on relevant information, we aimed to minimize the number of levels. A data structure with repeated measures for both timepoints and unstructured covariance structure was chosen based on mdodel fit criteria (log likelihood and corrected Akaike’s information criteria). Most fixed effects were held constant for both time points. For the Distress Thermometer, GAD-7, PHQ-9, and AUDIT-C, repeated measures scores were included in the analysis.

The initial model was fitted to estimate all fixed effects together in one model. An elimination by hand was applied, where predictors with fixed effect coeeficients having p-values above 0.10 were removed from the model. Only the final four predictors remained after this process. For the final model, a p-value of < 0.05 was used to declare statistical significance.

Because literature reported different drinking behavior post cancer-diagnosis for men and women, a gender*timepoint interaction term was included.

During model development, information criteria (including − 2 Restricted Log Likelihood and Corrected Akaike’s Information Criterion) were used to assess improvements in the model fit. Given a set of candidate models for the data, the model with the smallest values should be considered the best fit (see Table 2 for summary information criteria).

**Table 2:** Summary information criteria.

	−2 log likelihood	Akaike corrected	Bayesian
Model 1	2477,400	2483,442	2496,442
Model 2	2429,812	2435,744	2448,812

## Results

### Cohort characteristics

A total of 300 patients with cancer were included in the analysis. Due to the a priori definition for the EM and missing data in the GAD-7, 1.3% (*N* = 4) of the patients were excluded from the GLM. The descriptive statistics are pictured in Table 3.

**Table 3:** Sociodemographic and medical characteristics at baseline.

		Cancer patients (*N* = 300)			
		N			**%**
Age in years					
Mean (SD)		52.74 (12.95)			
18–29		17			5.7
30–49		92			30.7
50–69		162			54.0
70+		29			9.7
Gender					
Female		216			72.0
Male		84			28.0
Marital status					
Single		81			27.0
Married		169			56.3
Divorced		38			12.7
Widowed		12			4.0
Partnership					
In a partnership		203			67.7
Single		80			26.7
Other		17			5.7
Children					
Yes		124			41.3
No		176			58.7
Highest educational degree					
University or college degree		99			33.0
Technical college/ apprenticeship		179			59.7
No professional training		22			7.3
Occupation					
Working		175			58.3
Unemployed		18			6.0
Retired		79			26.3
Stay at home		11			3.7
Other		17			5.7
Currently on sick leave		158			52.7
Cancer type					
Female breast*		83			27.7
Urogenital		39			13.0
Gastrointestinal*		29			9.7
Hematological		26			8.7
Gynecological		21			7.0
Brain		18			6.0
Lung		14			4.7
Other		14			4.7
Melanoma		12			4.0
Lymphoma		12			4.0
Head-neck-cancer*		11			3.7
Endocrine		7			2.3
Sarcoma		5			1.7
Missing		9			3.0
Alcohol-associated cancer					
*Yes		128			42.7
No		172			57.3
Time since first diagnosis					
Mean years (SD)		2.74 (5.27)			
< 1 year		115			38.3
1–5 years		142			47.3
> 5 years		43			14.3
Treatment (multiple responses; past or present vs. not applicable, planned)					
Operation		203			67.7
Radiotherapy		103			34.3
Chemotherapy		164			54.7
Antihormonal		48			16.0
Stem cell therapy		18			6.0
Pain treatment		25			8.3
Number of comorbidities					
Mean (SD)		0.71 (0.99)			
Minimum		0			
Maximum		7			
0		159			53.0
1		95			31.7
2		26			8.7
3+		20			6.6
Use of psychopharmaceuticals		69			23.0

* SD *  Standard deviation.

*alcohol-associated cancers: head-and-neck-cancer, female breast and gastrointestinal cancer.

The majority of patients were female (*N* = 216, 72%) with a mean age of 52.74 years (SD 12.95). About half of the patients were married, a third were single, and other 10% were divorced.

Female breast cancer was the most common, accounting for about one-third of patients, followed by urogenital and gastrointestinal cancers. Approximately 40% of the patients suffered from alcohol-related cancers. The mean time since diagnosis was 2.74 years. Many patients received multimodal treatment including radiotherapy and chemotherapy (*N* = 393, 66.4%). The majority of patients (*N* = 203) underwent surgery (see Table 3).

### Psychosocial characteristics

Regarding alcohol consumption, the mean AUDIT-C sum score at baseline was 1.84 (SD = 1.76). The prevalence of abstainers was 29.3% at baseline and 30.7% at follow-up.

Almost half of the female patients (49.1%) and more than one third of the male patients (38.1%) showed risky drinking behavior. The highest AUDIT-C score reached at baseline was 8. During the 6-month follow-up, the mean AUDIT-C sum score decreased to 1.69 (SD = 1.65). At follow-up, the prevalence of women with risky drinking behavior decreased to 41.2%, while the prevalence of men increased up to 42.9%. The mean distress thermometer score was 7.02 (SD 1.9) at baseline and decreased to 5.84 (SD 2.34) at follow-up. Regarding anxiety symptoms the mean GAD-7 summary score was 10.37 at baseline and 7.71 at follow-up.

For depressive symptoms the mean PHQ-9 summary score was 11.16 (SD 5.81) at T0 and 8.49 (SD 5.49) at follow-up. For quality of life, the mean SF-8 score was 25.35 (SD 6.28). Quality of life scores in EORTC29 were 3.64 (SD 1.33) and in EORTC30 3.51 (SD 1.38), see Table 4.

**Table 4:** Psychosocial characteristics at baseline (T0) and follow-up (T1), *N* = 300.

	T0				T1		
	N		%		N		%
Distress thermometer							
Mean (SD)	7.02 (1.90)				5.84 (2.34)		
GAD-7							
Mean (SD)	10.37 (5.22)				7.71 (4.92)		
Missings					8		
PHQ-9							
Mean (SD)	11.16 (5.81)				8.49 (5.49)		
Missings					4		
SF-8							
Mean (SD)	25.35 (6.28)				n.a.**		
EORTC 29							
Mean (SD)	3.64 (1.33)				n.a.*		
EORTC 30							
Mean (SD)	3.51 (1.38)				n.a.*		
AUDIT-C sum score			%				%
Mean (SD)	1.84 (1.76)				1.69 (1.65)		
0	88		29.3		92		30.7
1	55		18.3		67		22.3
2–3	107		35.7		99		33.0
4+	50		16.7		42		14.0

*SD * Standard deviation, *n.a.* T1 not included the analysis, * Item only collected during the baseline survey, ** excluded for T1 due to a high number of missing data.

### Factors associated with alcohol consumption

#### Model 1

When all fixed effects were tested together in the first model, a significant association with risky alcohol consumption was found for the covariates number of comorbidities, anxiety symptoms (GAD-7), depressive symptoms (PHQ-9), as well as the interaction term (Gender*Timepoint) (see Table 5).

Patients with a higher number of comorbid conditions had significantly lower odds of engaging in risky drinking behavior. Each additional comorbidity was associated with approximately a 30% reduction in risk (OR = 0.704, 95% CI: 0.557–0.889). Second, higher levels of anxiety (GAD-7) were positively associated with risky alcohol consumption. For each additional point on the GAD-7 scale, the odds of risky drinking increased by 8.5% (OR = 1.085 (95% CI: 1.026–1.147). In contrast, higher levels of drepressive symptoms (PHQ-9) were associated with a small but significant reduction in risky drinking. Each unit increase in the PHQ-9 score was associated with about a 5.1% decrease in the odds of risky consumption (OR = 0.949, 95% CI: 0.902–0.999). Age showed a statistical trend towards significance (*p* = .109), suggesting a potential negative association with risky drinking (OR = 0.986, 95% CI: 0.968–1.003). As age was identified as a marginal predictor, the covariate was included in the second model.

**Fig. 2:**
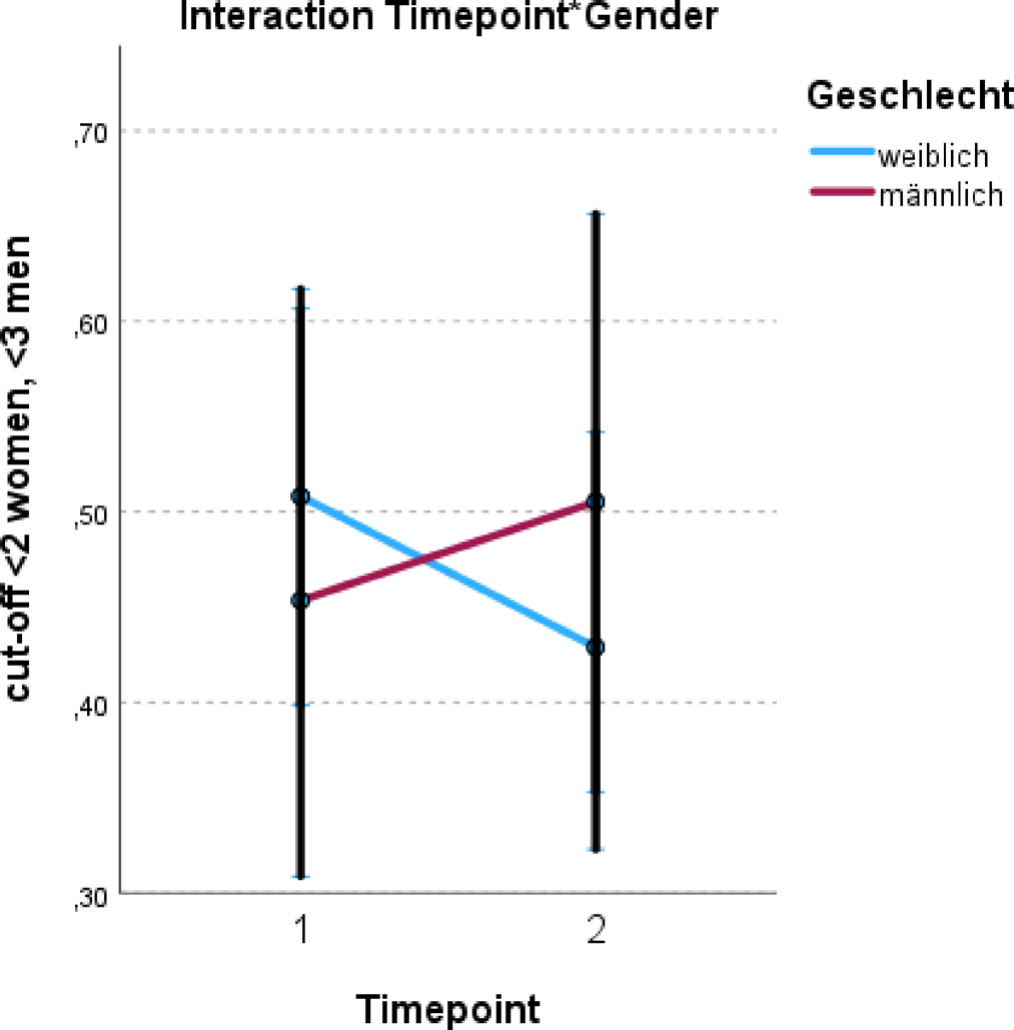
Interaction effect of timepoint and gender on risky alcohol use. Geschlecht = gender, weiblich = female, männlich = male (german translation).

Importantly, the interaction between gender and timepoint was also significant: female patients at T0 showed significantly higher odds of risky drinking compared to the reference group (male patients at T1; OR = 1.692, 95% ci: 1.024–2.798). This finding indicates that changes in risky alcohol use over time after a cancer diagnosis vary by gender. While female patients showed a decrease in risky drinking behavior at the 6-month follow-up, male padients displayed a slight increase (see Fig. 2).

**Table 5:** Model 1, fixed coefficients.

	*p*-value	Odds ratio (OR)	95% confidence interval for OR	
			Lower	Upper
Timepoint (T0)	0.380	0.,813	0.510	1.294
Gender (female)	0.296	0.736	0.413	1.310
Partnership (in a partnership)	0.681	0.802	0.278	2.309
Children (none)	0.267	1.310	0.812	2.114
Education status (lower)	0.287	1.628	0.662	4.005
Alcohol-associated cancer type (no)	0.484	0.841	0.517	1.368
Systemic therapy (radiation/ chemo/ stem cell therapy) (no)	0.780	1.071	0.661	1.736
Age	0.109	0.986	0.968	1.003
Overall health (EORTC_29)	0.280	0.867	0.669	1.123
Overall health (EORTC_30)	0.854	0.978	0.774	1.237
Number of comorbidities	**0.003**	0.704	0.557	0.889
Time since diagnosis	0.448	0.985	0.947	1.024
Health-related quality of life (SF-8)	0.367	0.975	0.923	1.030
Acute distress (DT)	0.440	0.964	0.877	1.059
Anxiety symptoms (GAD-7)	**0.004**	1.085	1.026	1.147
Depressive symptoms (PHQ-9)	**0.047**	0.949	0.902	0.999
Psychopharmaceuticals	1.000	1.000	0.562	1.781
Gender (female)*Timepoint (T0)	**0.040**	1.692	1.024	2.798

Binomial distribution with a logit link function; * p < .10;; dependent variable risky drinking behavior (‘group 2’).

#### Model 2

The second model, which includes only the remaining (marginally) significant variables after elimination by hand, provides a more focused analysis. The reduced complexity improves the model’s interpretability, focusing on key factors associated with risky alcohol drinking in patients with cancer over the six-month period. The improved information criteria suggest that excluding other irrelevant variables improved the predictive power of the model without compromising accuracy (see Table 2).

In the final model, a higher number of comorbidities, older age, and higher levels of depressive symptoms were identified as significant predictors of a lower likelihood of risky alcohol consumption (see Table 6). In contrast, higher anxiety levels were associated with an increased likelihood of engaging in risky drinking.

Consistent with the findings from Model 1, each additional comorbidity was linked to a 30% reduction in the odds of risky drinking behavior (OR = 0.707; 95% CI: 0.567–0.883). In contrast, for each one-point increase in the GAD-7, the odds of risky drinking increase by approximately 7.5% (OR = 1.075, 95% CI: 1.021–1.132). Higher levels of depressive symptoms were associated with reduced odds of risky drinking (OR = 0.952, 95% CI: 0.907–0.998). Age was also a significant predictor (OR = 0.983, 95% CI: 0.967–0.999), indicating that younger patients were more likely to engage in risky drinking behavior. Interaction terms between gender and timepoint were included in the model, but did not reach statistical significance. Thus, no gender-specific differences in drinking behavior over time following a cancer diagnosis could be confirmed.

**Table 6:** Model 2, fixed coefficients.

	*p*-value		95% confidence interval for OR	
		Odds ratio (OR)	Lower	Upper
Number of comorbidities	**0.002**	0.707	0.567	0.883
Anxiety symptoms (GAD-7)	**0.006**	1.075	1.021	1.132
Depressive symtoms (PHQ-9)	**0.040**	0.952	0.907	0.998
Age	**0.042**	0.983	0.967	0.999
Gender (female)*Timepoint (T0)	0.855	1.052	0.609	1.818

Binomial distribution with logit link function, dependent variable risky drinking behavior (‘Group 2’) **significance**: p-value < 0.05.

## Discussion

### Alcohol consumption patterns

The results indicate that alcohol drinking is common among psychologically distressed patients with cancer. Over a six-month period, about half of both female and male patients in treatment reported risky and potentially harmful drinking behaviors, both at the initial assessment and during follow-up.

Overall, there was no statistically significant difference in the prevalence of risky drinking between both timepoints, suggesting that a cancer diagnosis alone may not substantially alter patients’ alcohol consumption patterns. Likewise, other studies reported that up to 60% of cancer patients do not alter their drinking behavior after receiving a cancer diagnosis^35–37^. In previous research, up to 78% of patients with cancer were current drinkers and up to 60% engaged in risky drinking (e.g., > 1 drink for women and > 2 drinks for men)^31,32,47^. Notably, patients with cancer in other studies did not receive psycho-oncological care.

Studies have reported that the prevalence of risky alcohol drinking among patients with cancer is similar to that in non-cancer controls^34^. An important problem seems to be the lack of guidelines regarding alcohol consumption in patients with cancer.

Nevertheless, our results indicate gender-specific patterns in changes of risky alcohol use after cancer diagnosis. Although gender did not emerge as a statistically significant predictor for risky drinking behavior over the six-months period, differences in prevalence trends were observed: while female patients showed a decrease in risky drinking behavior at the six-month follow-up, male patients displayed a slight increase (T0: 49.1% of the female patients and 38.1% of the male patients; T1: 41.2% of the female and 42.9% of the male patients). Furthermore, the results of the analysis suggested that female patients were more likely to alter their drinking behavior after diagnosis.

Despite substantial evidence in the literature highlighting the detrimental effects of alcohol on cancer prognosis^11,14,15^, current data are insufficient to establish specific consumption levels that clearly worsen a cancer diagnosis or to define a precise threshold at which drinking becomes harmful^72^.

However, the revised recommendations of the German Nutrition Society^30^, coupled with the strong endorsement of complete abstinence by prominent organizations such as the World Cancer Research Fund^7^, send a clear message to all patients and their treating physicians: total abstinence from alcohol should be strongly encouraged and recommended^72^. Thus, the high number of patients with cancer who continue to drink alcohol after their cancer diagnosis is particularly concerning^7,37^.

In the absence of official guidelines on alcohol consumption for patients with cancer, we applied lowered cut-off scores for risky and potentially harmful drinking behavior for both genders, still accounting for gender differences in alcohol tolerance. More research is needed on the relationship between levels of alcohol consumption and the progression and worsening of cancer.

In our study population, the prevalence of abstainers increased only slightly over the six-month period, with an absolute difference of 1.4% between baseline and follow-up. These findings are consistent with previous studies, that reported that only 1.3% of patients stopped drinking after receiving their cancer diagnosis^26^. Interestingly, qualitative interviews with patients with cancer indicated that reductions in consumption were most often due to reduced tolerance to alcohol resulting from deteriorating health or cancer treatment, rather than a deliberate or conscious attempt to make healthy choices^18^. This discrepancy may be due to a lack of knowledge about the beneficial effects of reducing alcohol intake, as well as lack of awareness, motivation, or ability to reduce or stop drinking among psychologically distressed patients with cancer^17^.

Given that a cancer diagnosis has the potential to serve as a teachable moment for health behaviors^12,39,73,74^, our data suggest insufficient progress in patient education and the reduction of alcohol consumption among patients with cancer.

During the psycho-oncological treatment, alcohol consumption was not specifically targeted as a focus of intervention. Instead, patients were given the opportunity to talk about the psychological distress associated with their cancer diagnosis and to discuss coping strategies.

Although alcohol use was not directly addressed, it seems plausible that the psycho-oncological support may facilitate cognitive restructuring and psychoeducation, contributing to a shift towards a healthier lifestyle. Then again, patients might also feel healthier and less at risk. While cancer patients who undergo psycho-oncological treatment may be generally more burdened by psychological distress or unhealthy coping strategies^50,54,55^, the treatment could also pose a relevant chance for self-reflection, psycho-education, behavioral changes and support^57,58^. Interestingly, in our sample higher depressive symptoms were associated with reduced odds of alcohol consumption. Possible explanations are discussed below.

Psycho-oncological services have been associated with a significant reduction in distress and depressive symptoms among patients with cancer^50,54^. The high number of drinkers in psycho-oncological treatment, together with the clear evidence for a change in attitudes about the ‘harmlessness of alcohol’, highlight the need for a more integrated approach, where discussions about alcohol use are explicitly included in cancer care and also psycho-oncological support. All forms of alcohol consumption can be potentially risky for patients with cancer.

By addressing alcohol consumption directly, therapists could further enhance the effectiveness of their interventions and help patients to take a comprehensive approach to their health and well-being during cancer treatment.

Previous studies have suggested that participants scoring above 3 for women and 4 for men in the AUDIT-C are likely to be at high risk of hazardous drinking and alcohol dependence^75^. In our study cohort, approximately 17% of patients scored 4 points or more on the AUDIT-C at baseline, and 14% maintained this level of use at follow-up. Patients with potential alcohol dependence may have more difficulty to reduce or stop drinking, thus should be given special attention and support during screening and treatment.

### Predictor variables

In our sample, over the six-months period alcohol consumption was associated with the covariates number of comorbidities, depressive and anxiety symptoms, as well as age. These can be used as predictors variables for risky alcohol drinking behaviors in patients with cancer.

#### Number of comorbidities

Our data showed a negative association between the number of comorbidities and potentially harmful alcohol consumption. With each additional comorbidity, patients’ odds of risky drinking decreased significantly by about 30%. This is in contrast to the findings of previous researchers who reported significantly poorer health behaviors, such as healthy eating, physical activity, smoking, and alcohol consumption, and a lower adherence to healthy behaviors in patients with more comorbidities^26^. Patients with cancer with more comorbidities may have poorer overall health and lower tolerance for harmful substances, including alcohol.

They may also be taking more prescribed medications that are not recommended for use with alcohol, be more likely to experience adverse effects, or be advised to quit consumption more often. In addition, these patients may perceive themselves to be at higher risk and might therefore be more likely to make healthy behavioral changes.

#### Anxiety symptoms

In our sample, higher levels of anxiety were associated with an increased likelihood of risky drinking behavior, a finding consistent with previous research^52,53^. Reasons may be the initial stress-relieving and calming effects of alcohol^52,53^. With high levels of anxiety due to the disease burden or psychological distress^50,54^, patients may feel an increased need for relaxation and tension-relieving substances, such as alcohol. Notably, anxiety levels in our sample remained above the cut-off for relevant anxiety symptoms at both timepoints, with only a slight decrease over time, which may be attributed to the effects of psycho-oncological support. Our findings suggest that elevated anxiety symptoms in patients with cancer may contribute to increased alcohol use as a maladaptive coping strategy. These patterns highlight the importance of addressing anxiety in psycho-oncological care and promoting healthier coping mechanisms to reduce the risk of harmful drinking.

#### Depressive symptoms

As mentioned above, reduced odds of risky drinking were observed for higher levels of depressive symptoms. On the contrary, alcohol use and abuse were significantly associated with depressive symptoms in patients with cancer in other studies^35,51^, while others could not find an association between depressive symptoms and alcohol consumption^53,76^. It was expected that by reducing symptoms of depression and helping patients develop healthier coping mechanisms, the psycho-oncology care may have positively impacted alcohol use and fostered healthy behaviors^50,54^. Then again, patients with lower levels of depressive symptoms may perceive themselves as being at lower risk and therefore see fewer reasons to change their behavior post-diagnosis, continuing pre-existing drinking habits without recognizing potential health risks.

They may feel more emotionally stable and be more socially active, leading to more frequent exposure to alcohol-related social situations^77^ and a higher likelihood of a normalized routine including drinking. Conversely, patients with higher depressive symptoms may lead more socially withdrawn lives or experience poorer health, making them less likely or physically unable to tolerate alcohol.

For depressive symptoms, the average PHQ-9 scores fell below the cut-off for relevant depressive symptoms from baseline to follow-up, although significance was not tested. Similarly, previous studies have also supported a significant reduction in depressive symptoms for patients who receive psycho-oncological support^54^.

#### Age

 The results of this analysis showed a decrease in alcohol consumption with increasing age. This is consistent with previous studies showing an association between older age and lower odds of being a current drinker and high-risk or heavy drinking^31,47^. The decrease in consumption has been attributed to, among other things, increased social or family responsibilities in older patients^43,78^. Our cohort included a relatively high number of younger patients, with one third between 30 and 49 years. Plausible contributors are several factors such as university hospital, larger city, psycho-oncology treatment, and cancer type (most common: female breast cancer). On the other hand, high-frequency drinking was more prevalent in older patients in former research^16^. Due to the limited sensitivy of the short form AUDIT-C, the scores, particularly in the midrange, do not help to clearly differentiate between light but regular and infrequent drinking with higher quantities per occasion.

### Variables not associated with alcohol consumption

As mentioned above, timepoint did not show a significant association with risky drinking behavior. Consequently, no significant difference in the overall prevalence of risky drinking behavior was found between both timepoints. The lack of a time-related effect may suggest that reeiving a cancer diagnosis does not necessarily lead to a reduction in alcohol consumption and challenges the concept of the teachable moment^12^.

Likewise, gender could not be established as a predictor for risky drinking, even though results indicated that the effect of timepoint on risky drinking behavior varied by gender. A large number of studies report significantly lower levels or alcohol drinking for women and higher likelihood of reducing or quitting drinking compared to men^43,45^. In the first model, female patients showed a decrease in risky drinking behavior at the six-month follow up, while male patients showed a slight increase, but the association could not be established in the final model calculation. Then again, other researchers also found that contined drinking after a cancer diagnosis was not influenced by gender^35,42^.

*Cancer-related factors*, including *time since diagnosis*,* type of cancer*,* and cancer treatment*, showed no significant association with alcohol consumption in our study. Previous research suggested that patients with cancer are more likely to adopt positive health behaviors shortly after diagnosis^15,79^, yet our analysis did not support an association between longer survival time and current drinking^36^. Additionally, alcohol consumption did not differ significantly between patients with alcohol-related cancers and those with other types of cancer in our study. Variations in drinking behavior observed in patients undergoing different cancer treatments, such as radiation or chemotherapy, may be attributed to factors like impaired oral function and alcohol tolerance^42^. Possible reasons why cancer treatment did not emerge as a significant predictor include limited variability within the treatment variable, potential ceiling effects from high psycho-oncological distress, or confounding with other variables.

*Sociodemographic variables* such as *educational status*,* having children*,* and partnership* did not show an association with alcohol consumption in our study. Previous research has shown conflicting findings regarding education, with some studies linking a lower education to higher likelihood of risky alcohol consumption and others reporting high prevalence of hazardous drinking among highly educated individuals^26,37,48^. The skewed distribution of education in our cohort (93% with continuing education) may explain the lack of significance. In Germany, free access to support services like psycho-oncology treatment may provide equal opportunities for all patients to make health-conscious choices.

Parenthood and partnership did not reduce alcohol consumption as suggested by previous non-cancer population studies^31,49^, suggesting a need for further investigation into the influence of children on parental drinking behavior that may include information about the number of children, their ages, and living together in the same household.

Regarding *psychosocial variables*, no association was found for *quality of life*,* distress*, and *use of psychopharmaceuticals* in this analysis.

The association of better quality of life or distress scores (SF-8, EORTC, DT) and higher levels of alcohol consumption, as suggested in previous research^15,31,35^ could not be supported by this analysis.

Notably, the poor scores on measures of quality of life, well-being, and distress in this patient sample indicate high levels of psychosocial and physical distress. These scores may have acted as ceiling effects, limiting the ability to explain the variability in alcohol consumption.

### Limitations and strengths

When discussing the results of this study, some limitations must also be considered: as this study is limited to self-reported data, recall or classification bias must be considered.

Due to social desirability, patients may underreport their alcohol consumption in the questionnaire^80,81^. Previous research has suggested that patients who do not respond to the questions are more likely to be heavy drinkers^64^. The accuracy of patients’ understanding of quantities and portions must also be questioned, although detailed information on standard sizes was provided in the questionnaires.

Despite these limitations, previous research has shown that the AUDIT-C is a well-accepted screening tool for alcohol consumption that does not elicit strong resistance from participants^82^. As this was a secondary analysis, patients were not specifically informed in advance that information about their drinking patterns would be collected. This may have distracted patients from evaluating their drinking and may reduce the desire to “look good” or perform well.

The analysis was limited to patients with cancer receiving outpatient psycho-oncology treatment.

Further research should also include a control group of either patients with cancer without psycho-oncological support, non-cancer control groups, or even family members, or collect data on drinking patterns before and after diagnosis to further analyze the influence of the cancer diagnosis on alcohol consumption. Using a control group, the impact of psycho-oncology support on patients’ alcohol use should be further investigated, but again, alcohol consumption was not systematically addressed in the consultations. Another notable limitation of our study is the potential selection bias due to participant drop-out in this secondary analysis of real-life data. Reasons for drop-out included high physical and psychological burden of the disease, lack of interest, and death.

These factors may have influenced the representativeness of our longitudinal sample and should be considered when interpreting the results. In particular, it is possible that patients with more severe illness or higher distress levels were less likely to complete the follow-up adessment, which could lead to an underestimation of psychological burden or risky behaviors such as alcohol use.

Another limitation is that tumor staging could not be included in the analysis, due to missing data. This is significant because an advanced tumor stage or the presence of metastases might be associated with changes in alcohol consumption. On the one hand, disease progression or metastasis could lead to increased alcohol use as a coping mechanism. On the other hand, it is also possible that patients at these stages may reduce their alcohol consumption to avoid further compromising their health.

A number of patients had to be excluded due to missing data. However, we were still able to reach a relevant number of participants to analyze our research questions with sufficient statistical power.

Finally, information on tobacco use was not collected in the questionnaire. Previous studies have shown that alcohol consumption, especially high-risk drinking, is more common in patients who smoke^31,47,83,84^. In addition, the combination of alcohol and tobacco has a synergistic multiplicative effect on the risk of developing cancer or a second primary cancer, as well as negative effects on treatment^85,86^.

In addition, other research has shown that former and current smokers are more likely to be current drinkers and exceed moderate or binge drinking than never smokers^32^.

On the other hand, patients who use both alcohol and tobacco may have more difficulty making behavioral changes and may need to be specifically targeted in treatment^42^. Further studies are needed to determine whether smoking could be a predictor of risky alcohol consumption.

Overall, this longitudinal analysis, which included patients with cancer with a variety of cancer types, tumor stages, and cancer treatment modalities, provided a valuable contribution to a better understanding of the health behaviors, psychosocial conditions, and alcohol use of distressed patient with cancers in outpatient psycho-oncology treatment.

Previous studies have often focused on a specific type of cancer, and it is likely that patients with the same type of cancer share other characteristics or health behaviors that influence their drinking patterns, leading to results that are not generally applicable to patient with cancers.

## Conclusion

Similar to previous studies, we found that the majority of patients with cancer are current drinkers and continue drinking after receiving a cancer diagnosis^31,32^. Overall, alcohol consumption did not decrease over the six-month period. The lack of progress regarding drinking behaviors after a cancer diagnosis, indicates insufficient progress in patient care and education. The generally high number of patients who consume alcohol after a cancer diagnosis, despite the clear recommendation of abstinence for patients with cancer, underscores the need for action.

In this population of psychologically distressed patients with cancer, having fewer comorbidities, higher levels of anxiety, lower levels of depressive symptoms, and being younger were predictors of risky and potentially harmful alcohol consumption.

Our findings have important implications for improving the development of better screening and assessment of alcohol use in patients with cancer within psycho-oncology treatment, helping to identify those at risk of increased drinking who therefore require special monitoring throughout their survival period.

One of the main challenges appears to be the lack of appropriate and widely accepted guidelines for alcohol consumption specific to patients with cancer and survivors^15^. As the AUDIT-C questionnaire is a commonly used assessment tool in research, the threshold for risky drinking behavior in patients with cancer must be reconsidered and more research is needed to provide precise information about the amount of alcohol intake that can deteriorate a cancer prognosis.

According to the current state of research, patient education and targeted guidelines for patients with cancer should include the clear advice to avoid any alcohol consumption.

It is expected that patients who have a better understanding of the relationship between alcohol and cancer will be more willing to make positive and even uncomfortable changes in their health behaviors. Targeted assessment of drinking behavior, patient education, and support for cessation could be incorporated into psycho-oncology treatment.

Predictors of risky drinking can be used to target patients with cancer at risk for excessive alcohol consumption.

## Electronic supplementary material

Below is the link to the electronic supplementary material.

Supplementary Material 1

Supplementary Material 2

## Data Availability

This publication contains all relevant data. Detailed data will be made available upon demand, e.g. for systematic reviews or meta-analyses.

## Abbreviations

AICR/WCRF: American Institute for Cancer Research/World Cancer Research Fund
AUDIT: Alcohol use disorders identification test
AUDIT-C: Alcohol use disorders identification test- consumption
EORTC: European organisation for treatment of cancer
EORTC QLQ-C30: EORTC quality of live questionnare core 30
GAD-7: Generalized anxiety disorder scale-7
HRQoL: Health related quality of life
IARC: International agency for research on cancer
ICD-10: International statistical classification of diseases and health problems
NCCN: National comprehensive cancer network
NCCN DT: NCCN distress thermometer
PHQ-9: Patient health questionnaire-9
QoL: Quality of life
RR: Correlation coefficient
SD: Standard deviation
SF-36: Short-form-36 health survey
SF-8: Short-form-8 health survey
UKE: University Medical Center Hamburg Eppendorf
WHO: World Health Organization
