# Supplementary material for: A longitudinal analysis on alcohol consumption in patients with cancer undergoing psycho-oncological treatment
Source: Sci Rep. 2025 Jun 20;15:20137. doi: 10.1038/s41598-025-04849-w (PMC12181269; doi:10.1038/s41598-025-04849-w)
Supplement: Supplementary file 1 — Supplementary Material 1 [file 41598_2025_4849_MOESM1_ESM.doc]

**Questionnaire on**

**psychosocial stress**

Patient questionnaire

**A. Personal Data**

| **Today’s date** | | | | Day | | | | | Month | | Year | | **Age** | | ……... | | | | **Gender** | | | | | | | 🔿 female |
| --- | --- | --- | --- | --- | --- | --- | --- | --- | --- | --- | --- | --- | --- | --- | --- | --- | --- | --- | --- | --- | --- | --- | --- | --- | --- | --- |
|  | | | |  | | |  | |  |  |  |  |  | |  | | | |  | | | | | | | 🔿 male |
| 1. | **Marital status** | | | |  | | | | | | | | | **Do you have…** | | | | | | | |  | | | | |
|  | 🔿 | single | | | |  | |  | | | | | | 🔿 | in a partnership | | | | | | | | | | | |
|  | 🔿 | married | | | |  | |  | | | | | | 🔿 | alone | | | | |  |  | | | | | |
|  | 🔿 | divorced | | | |  | |  | | | | | | 🔿 | other ……………………………. | | | | | | | | | | | |
|  | 🔿  🔿 | registered civil partnership  widowed | | | | | | | | | | | |  |  | | | | |  |  | | | | | |
| 2. | **Do you have children? yes** 🔿 **no 🔿** | | | | | | | | | | | | | | | | | | | | | | | | | |
|  | If you have children, how many children do you have? ___________ (number) | | | | | | | | | | | | | | | | | | | | | | | | | |
| **Year of birth Gender Does this child live in your household?** | | | | | | | | | | | | | | | | | | | | | | | | | | |
| male female yes no  1) 🔿 🔿 🔿 🔿  2) 🔿 🔿 🔿 🔿  3) 🔿 🔿 🔿 🔿  4) 🔿 🔿 🔿 🔿 | | | | | | | | | | | | | | | | | | | | | | | | | | |
| 3. | **What is your highest school-leaving qualification?** | | | | | | | | | | | | | | | | | | | | | | |  | | |
|  | 🔿 | elementary or secondary school certificate | | | | | | | | | | | | | | 🔿 Abitur (university entrance qualification) | | | | | | | | | | |
|  | 🔿 | secondary school leaving certificate | | | | | | | | | | | | | | 🔿 other/no school-leaving qualification | | | | | | | | | | |
|  | 🔿 | advanced technical college certificate | | | | | | | | | | | | | | ……………………… | | | | | | | | | | |
| 4. | **What vocational training did you complete?** | | | | | | | | | | | | | | | | | | | | | | | | | |
|  | 🔿 | apprenticeship (vocational/company training) | | | | | | | | | | | | | | | | | | | | | | | | |
|  | 🔿 | technical school (master/technical school, vocational/technical academy) | | | | | | | | | | | | | | | | | | | | | | | | |
|  | 🔿 | technical college/engineering school | | | | | | | | | | | | | | | | | | | | | | | | |
|  | 🔿 | university/college | | | | | | | | | | | | | | | | | | | | | | | | |
|  | 🔿 | other/no vocational training ………………………………………………………. | | | | | | | | | | | | | | | | | | | | | | | | |
| 5. | **What is your current work situation?** | | | | | | | | | | | | | | | | | | | | | | | | | |
|  | 🔿 | employed; full-time | | | | | | | | | | | | | | | | | | | | | | | | |
|  | 🔿 | employed; part-time | | | | | | | | | | | | |  | | |  | | | |  | | | | |
|  | 🔿 | unemployed since ________________ | | | | | | | | | | | | | | | | | | | | | | | | |
|  | 🔿 | retired/retired since ________________  ***If retired:*** 🔿 due to age  🔿 reduced earning capacity pension  🔿 early retirement, but no reduced earning capacity pension | | | | | | | | | | | | | | | | | | | | | | | | |
|  | 🔿 | housewife/househusband | | | | | | | | | | | | | | | | | | | | | | | | |
|  | 🔿 | Other ………………………………………… | | | | | | | | | | | | | | | | | | | | | | | | |
| 6. | **Are you currently on sick leave (unable to work)?** | | | | | | | | | | | | | | | | | | | | | | | | | |
|  | 🔿 | | no | | | | | | | | | | | | month | | | | year | | | |  | | | |
|  | 🔿 | | yes **since when?** | | | | | | | | | | | |  | |  | |  | | |  | | |  | |

*-5-*

**B. Questions about the disease and treatment**

| **1.** | **What type of cancer have you been diagnosed with?** ………………………………….. | | | | | | | | | | |
| --- | --- | --- | --- | --- | --- | --- | --- | --- | --- | --- | --- |
| 2. | **When was the first diagnosis?** | | | | | | | | | | |
|  |  | | Date of diagnosis: | month | | year | | |  | | |
|  |  | |  |  |  |  |  | |  | | |
| 3. | What treatments did you undergo or are you undergoing? | | | | | | | | | | |
| What treatments have you undergone or are undergoing? |  |  | | | | | | **completed** | | **ongoing** | **planned** |
|  | 🔿 | surgery | | | | | | 🔿 | | 🔿 | 🔿 |
|  | 🔿 | radiotherapy | | | | | | 🔿 | | 🔿 | 🔿 |
|  | 🔿 | chemotherapy | | | | | | 🔿 | | 🔿 | 🔿 |
|  | 🔿 | anti-hormonal therapy | | | | | | 🔿 | | 🔿 | 🔿 |
|  | 🔿 | stem cell therapy | | | | | | 🔿 | | 🔿 | 🔿 |
|  | 🔿 | other therapies (antibodies, interferon...) | | | | | | 🔿 | | 🔿 | 🔿 |
|  | 🔿 | pain therapy | | | | | | 🔿 | | 🔿 | 🔿 |
|  | 🔿 | *other therapy ……………………………* | | | | | | 🔿 | | 🔿 | 🔿 |
| 4. | **Do you also have one or more of the diseases listed in the following?** | | | | | | | | | | |
|  | 🔿 | heart disease and circulatory disorders | | | | | | | | | |
|  | 🔿 | diseases of the nervous system | | | | | | | | | |
|  | 🔿 | diseases of the musculoskeletal system and inflammatory diseases | | | | | | | | | |
|  | 🔿 | diseases of the respiratory tract | | | | | | | | | |
|  | 🔿 | stomach disease/diseases of the digestive system | | | | | | | | | |
|  | 🔿 | bladder and kidney diseases | | | | | | | | | |
|  | 🔿 | hormonal diseases (e.g. diabetes) | | | | | | | | | |
|  | 🔿 | skin diseases | | | | | | | | | |
|  | 🔿 | other ………………………………………………………………………………………… | | | | | | | | | |
| 5. | **Are you currently receiving psychological or psychiatric treatment?** | | | | | | | | | | |
|  | yes 🔿 no 🔿 | | | | | | | | | | |
|  | **If yes, who is your treatment provider?** | | | | | | | | | | |
|  | ………………………………………………………………………………………………………... | | | | | | | | | | |

**C. Questions about registration in the special outpatient clinic for psycho-oncology**

| **1. On whose initiative did you register at the special outpatient clinic for psycho-oncology?** |
| --- |

| *✍ Please check* ***all*** *that apply:* | | **no** | **yes** |
| --- | --- | --- | --- |
| a. | I became aware of the *special outpatient clinic for psycho-oncology* myself and registered. | 🔿 | 🔿 |
| b. | My attending physician recommended that I register with the *special outpatient clinic for psycho-oncology.* | 🔿 | 🔿 |
| c. | My partner has recommended that I register with the *special psycho-oncology outpatient clinic.* | 🔿 | 🔿 |
| d. | Friends or acquaintances have recommended that I register with the *psycho-oncology outpatient clinic.* | 🔿 | 🔿 |
| e. | During rehabilitation ("cure"), I was recommended to register at the *special outpatient clinic for psycho-oncology.* | 🔿 | 🔿 |
| f. | Other  ……………………………………………………………………………………… | 🔿 | 🔿 |

| **2. I am interested in the following offers of the special outpatient clinic for psycho-oncology:** |
| --- |

| *✍ Please check* ***all*** *that apply:* | |  |  |
| --- | --- | --- | --- |
| a. | Supportive counseling sessions | 🔿 | |
| b. | Individual psychotherapeutic sessions | 🔿 | |
| c. | Group offers with other affected persons and/or relatives | 🔿 | |
| d. | Couples counseling | 🔿 | |
| e. | Art therapy (see additional information p.15 | 🔿 | |
| f. | Music therapy (see additional information p.15) | 🔿 | |
| g. | Parent and child-related support | 🔿 | |
| h. | Other  ………………………………………………………………………………………… | 🔿 | |

| **3. Questions about psychiatric and drug treatment** |
| --- |

| *✍ Please check* ***all*** *that apply:* | | **no** | **yes** |
| --- | --- | --- | --- |
| a. | Have you ever received outpatient psychiatric treatment? | 🔿 | 🔿 |
| b. | Have you ever received inpatient psychiatric treatment? | 🔿 | 🔿 |
| c. | Have you ever received inpatient psychosomatic treatment? | 🔿 | 🔿 |
| d. | Are you currently taking medication for mental health problems? | 🔿 | 🔿 |
|  | ***If yes,*** which medication do you take for mental health problems? | | |
|  | 🔿 antidepressants, name  …………………………………………………………………………………….  🔿 benzodiazepines/sleeping pills, name  …………………………………………………………………………………….  🔿 neuroleptics, name  ……………………………………………………………….…………………… | | |
|  | 🔿 other  ……………………………………………………………….………………….. | | |

**D. Questions about current stresses**


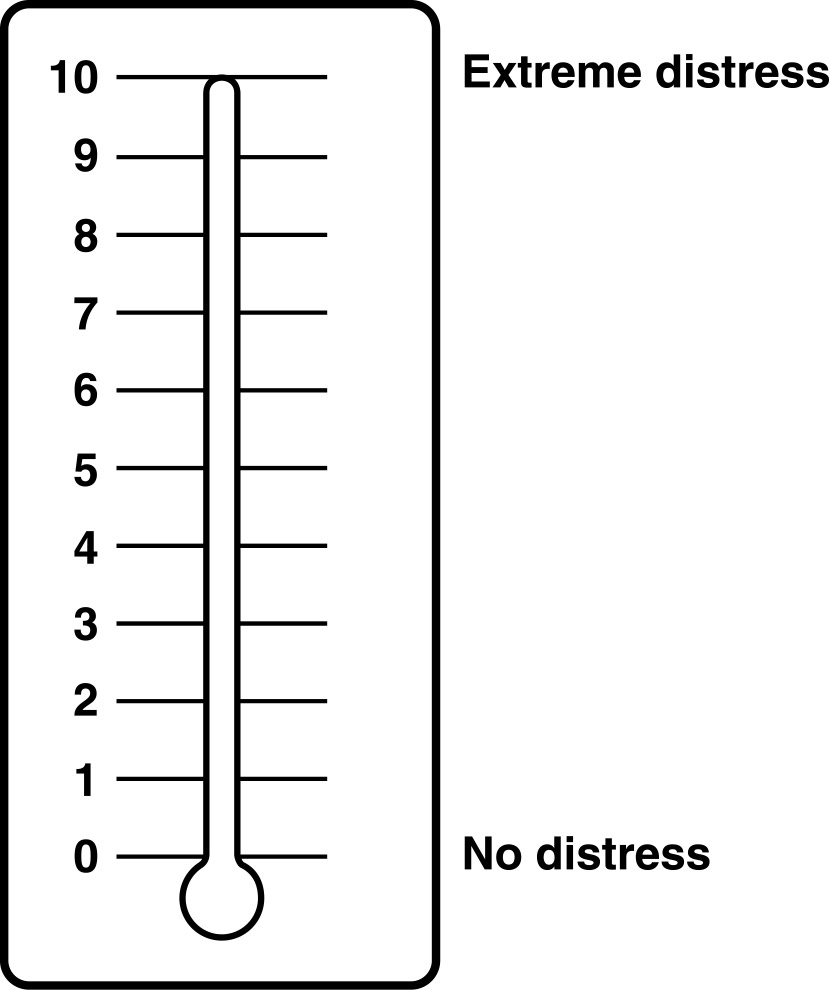


Extremely stressed

No distress

**Instructions:**

**1.** Please circle on the thermometer

the number on the right (0-10) that best describes

how stressed you have felt **in the last week**

**including today.**

| **2.** Please indicate whether you have had problems in any of the following areas in the last week, including today. Check **YES** or **NO** for each area. | | | | | | |
| --- | --- | --- | --- | --- | --- | --- |
|  | | | | | | |
| **YES** | **NO** |  |  | **YES** | **NO** |  |
|  |  | **Practical problems** |  |  |  | **Physical problems** |
| 🔿 | 🔿 | housing situation |  | 🔿 | 🔿 | pain |
| 🔿 | 🔿 | insurance |  | 🔿 | 🔿 | nausea |
| 🔿 | 🔿 | work/ school |  | 🔿 | 🔿 | exhaustion |
| 🔿 | 🔿 | transportation |  | 🔿 | 🔿 | sleep |
| 🔿 | 🔿 | childcare |  | 🔿 | 🔿 | movement/ mobility |
|  |  |  |  | 🔿 | 🔿 | washing, dressing |
|  |  | **Family problems** |  | 🔿 | 🔿 | appearance |
| 🔿 | 🔿 | dealing with your partner |  | 🔿 | 🔿 | breathing |
| 🔿 | 🔿 | dealing with your children |  | 🔿 | 🔿 | inflammation in the mouth |
|  |  |  |  | 🔿 | 🔿 | eating/ nutrition |
|  |  | **Emotional problems** |  | 🔿 | 🔿 | indigestion |
| 🔿 | 🔿 | worries |  | 🔿 | 🔿 | constipation |
| 🔿 | 🔿 | fears |  | 🔿 | 🔿 | diarrhea |
| 🔿 | 🔿 | sadness |  | 🔿 | 🔿 | changes in urination |
| 🔿 | 🔿 | depression |  | 🔿 | 🔿 | fever |
| 🔿 | 🔿 | nervosity |  | 🔿 | 🔿 | dry/ itchy skin |
| 🔿 | 🔿 | loss of interest in |  | 🔿 | 🔿 | dry/ stuffy nose |
|  |  | everyday activities |  | 🔿 | 🔿 | tingling in hands/ feet |
|  |  |  |  | 🔿 | 🔿 | feeling swollen/ puffy |
|  |  | **Spiritual/ religious matters** |  | 🔿 | 🔿 | memory/ concentration |
| 🔿 | 🔿 | in relation to God |  | 🔿 | 🔿 | sexual problems |
| 🔿 | 🔿 | loss of faith |  |  |  |  |
|  |  |  |  |  |  |  |
| **Other problems**  ………………………………………………………………………………………………………………………. | | | | | | |

| **3. To what extent have you felt affected by the following complaints in the last 2 weeks?** | | | | | |
| --- | --- | --- | --- | --- | --- |
|  |  | **not at all** | **on individual days** | **on more than half of all days** | **almost every day** |
| 1. | Nervousness, anxiety, or tension | 🔿 | 🔿 | 🔿 | 🔿 |
| 2. | Not being able to stop or control worries | 🔿 | 🔿 | 🔿 | 🔿 |
| 3. | Excessive worry about various matters | 🔿 | 🔿 | 🔿 | 🔿 |
| 4. | Difficulty relaxing | 🔿 | 🔿 | 🔿 | 🔿 |
| 5. | Restlessness, making it difficult to sit still | 🔿 | 🔿 | 🔿 | 🔿 |
| 6. | Quick anger or irritability | 🔿 | 🔿 | 🔿 | 🔿 |
| 7. | Feeling anxious, as if something bad is going to happen | 🔿 | 🔿 | 🔿 | 🔿 |

| **4. To what extent have you felt affected by the following complaints in the last 2 weeks?** | | | | | |
| --- | --- | --- | --- | --- | --- |
|  |  | **not at all** | **on individual days** | **on more than half of all days** | **almost every day** |
| 1. | Little interest or pleasure in activities | 🔿 | 🔿 | 🔿 | 🔿 |
| 2. | Depression, melancholy, or hopelessness | 🔿 | 🔿 | 🔿 | 🔿 |
| 3. | Difficulty falling asleep or staying asleep, or increased sleep | 🔿 | 🔿 | 🔿 | 🔿 |
| 4. | Tiredness or feeling of having no energy | 🔿 | 🔿 | 🔿 | 🔿 |
| 5. | Decreased appetite or excessive need to eat | 🔿 | 🔿 | 🔿 | 🔿 |
| 6. | Poor opinion of self; feeling like a failure or having let the family down | 🔿 | 🔿 | 🔿 | 🔿 |
| 7. | Difficulty concentrating on something, e.g. reading the newspaper, or watching TV | 🔿 | 🔿 | 🔿 | 🔿 |
| 8. | Were your movements or speech so slowed down that others would notice? Or, on the contrary, were you "fidgety" or restless and therefore had a stronger urge to move than usual? | 🔿 | 🔿 | 🔿 | 🔿 |
| 9. | Thoughts that you would rather be dead or want to harm yourself | 🔿 | 🔿 | 🔿 | 🔿 |

| **5. Please answer the following questions about your state of health.** |
| --- |

| **1. How would you generally describe your state of health in the past week?** | | | | | | | | | | | | | |
| --- | --- | --- | --- | --- | --- | --- | --- | --- | --- | --- | --- | --- | --- |
| excellent  🔿 | | Very good  🔿 | | good  🔿 | | | less good  🔿 | | | | bad  🔿 | | |
| **2. How much have problems with your physical health restricted you in the past week from normal physical activities (walking, climbing stairs)?** | | | | | | | | | | | | | |
| not at all  🔿 | | very little  🔿 | | moderately  🔿 | | | quite  🔿 | | | | was not able to do physical activities  🔿 | | |
| **3. To what extent did your physical health make it difficult for you to carry out your daily work at home and outside the home?** | | | | | | | | | | | | | |
| not at all  🔿 | | a little  🔿 | | moderately  🔿 | | | quite  🔿 | | | | was not able to do every day work  🔿 | | |
| **4. How severe was your pain in the past week?** | | | | | | | | | | | | | |
| I had no pain  🔿 | Very slight  🔿 | | slight  🔿 | | moderate  🔿 | | | | severe  🔿 | | | strong  🔿 | |
| **5. How much energy did you have in the past week?** | | | | | | | | | | | | | |
| very much  🔿 | | quite a lot  🔿 | | moderate  🔿 | | | a little  🔿 | | | | none  🔿 | | |
| **6. How much have your physical health or emotional problems in the past week affected your normal contacts with family members or friends in the past week?** | | | | | | | | | | | | | |
| not at all  🔿 | | very little  🔿 | | moderately  🔿 | | | quite  🔿 | | | | was not able to make this contact  🔿 | | |
| **7. How much have mental problems (e.g. anxiety, depression or irritability) bothered you in the past week?** | | | | | | | | | | | | | |
| not at all  🔿 | | very little  🔿 | | moderately  🔿 | | | quite a bit  🔿 | | | | very much  🔿 | | |
| **8. How much have your personal or emotional problems prevented you from carrying out your normal activities at work, school/study or other?** | | | | | | | | | | | | | |
| not at all  🔿 | | very little  🔿 | | moderately  🔿 | | | quite  🔿 | | | | was not able to do so  🔿 | | |
| **6. Please answer the following questions about your health by ticking which statement best applies to you.** | | | | | | | | | | | | | |
| **During the last week:** | | | | | | **not at all** | | **little** | | **moderately** | | | **very** |
| 1. Do you find it difficult to exert yourself physically (e.g. carry a heavy shopping bag or suitcase)? | | | | | | 🔿 | | 🔿 | | 🔿 | | | 🔿 |
| 2. Do you find it difficult to take a longer walk? | | | | | | 🔿 | | 🔿 | | 🔿 | | | 🔿 |
| 3. Do you find it difficult to walk a short distance outside of the house? | | | | | | 🔿 | | 🔿 | | 🔿 | | | 🔿 |
| 4. Do you have to lie in bed or sit in an armchair during the day? | | | | | | 🔿 | | 🔿 | | 🔿 | | | 🔿 |
| 5. Do you need help eating, dressing, washing, or using the toilet? | | | | | | 🔿 | | 🔿 | | 🔿 | | | 🔿 |
| **During the last week:** | | | | | | **not at all** | | **little** | | **moderately** | | | **very** |
| 6. Have you been restricted in your work or other daily activities? | | | | | | 🔿 | | 🔿 | | 🔿 | | | 🔿 |
| 7. Were you restricted in your hobbies or other leisure activities? | | | | | | 🔿 | | 🔿 | | 🔿 | | | 🔿 |
| 8. Were you short of breath? | | | | | | 🔿 | | 🔿 | | 🔿 | | | 🔿 |
| 9. Were you in pain? | | | | | | 🔿 | | 🔿 | | 🔿 | | | 🔿 |
| 10. Did you have to rest? | | | | | | 🔿 | | 🔿 | | 🔿 | | | 🔿 |
| 11. Did you have trouble sleeping? | | | | | | 🔿 | | 🔿 | | 🔿 | | | 🔿 |
| 12. Did you feel weak? | | | | | | 🔿 | | 🔿 | | 🔿 | | | 🔿 |
| 13. Did you have a lack of appetite? | | | | | | 🔿 | | 🔿 | | 🔿 | | | 🔿 |
| 14. Did you feel nauseous? | | | | | | 🔿 | | 🔿 | | 🔿 | | | 🔿 |
| 15. Did you vomit? | | | | | | 🔿 | | 🔿 | | 🔿 | | | 🔿 |
| 16. Were you constipated? | | | | | | 🔿 | | 🔿 | | 🔿 | | | 🔿 |
| 17. Did you have diarrhea? | | | | | | 🔿 | | 🔿 | | 🔿 | | | 🔿 |
| 18. Were you tired? | | | | | | 🔿 | | 🔿 | | 🔿 | | | 🔿 |
| 19. Did pain interfere with your daily life? | | | | | | 🔿 | | 🔿 | | 🔿 | | | 🔿 |

| **During the last week:** | | | | | **not at all** | | **little** | | **moderately** | | | **very** |
| --- | --- | --- | --- | --- | --- | --- | --- | --- | --- | --- | --- | --- |
| 20. Did you have difficulty concentrating on something, e.g. reading the newspaper, or watching television? | | | | | 🔿 | | 🔿 | | 🔿 | | | 🔿 |
| 21. Did you feel tense? | | | | | 🔿 | | 🔿 | | 🔿 | | | 🔿 |
| 22. Were you worried? | | | | | 🔿 | | 🔿 | | 🔿 | | | 🔿 |
| 23. Were you irritable? | | | | | 🔿 | | 🔿 | | 🔿 | | | 🔿 |
| 24. Did you feel depressed? | | | | | 🔿 | | 🔿 | | 🔿 | | | 🔿 |
| 25. Did you have difficulty remembering things? | | | | | 🔿 | | 🔿 | | 🔿 | | | 🔿 |
| 26. Has your physical condition or medical treatment affected your family life? | | | | | 🔿 | | 🔿 | | 🔿 | | | 🔿 |
| 27. Has your physical condition or medical treatment affected your social life or activities with other people? | | | | | 🔿 | | 🔿 | | 🔿 | | | 🔿 |
| 28. Has your physical condition or medical treatment caused you financial difficulties? | | | | | 🔿 | | 🔿 | | 🔿 | | | 🔿 |
|  | | | | | | | | | | | | |
| **For the following questions, please mark the number between 1 and 7 that best applies to you.** | | | | | | | | | | | | |
| 29. How would you rate your overall state of health during the last week? | | | | | | | | | | | | |
| 1 | 2 | | 3 | 4 | | 5 | | 6 | | | 7 | |
| **very bad** | |  | | | | | | | | **excellent** | | |
| 30. How would you rate your overall quality of life over the past week? | | | | | | | | | | | | |
| 1 | 2 | | 3 | 4 | | 5 | | 6 | | | 7 | |
| **very bad** | |  | | | | | | | | **excellent** | | |

**E. Questions about alcohol consumption**

| Some people try to cope with their problems, pain, and/or other stresses by self-medication, e.g. drinking alcohol.  **Please answer the following questions:** |
| --- |

| **1. How often do you drink alcohol?** | | |
| --- | --- | --- |
| a. | never | 🔿 |
| b. | once a month or less | 🔿 |
| c. | two to four times a month | 🔿 |
| d. | two to three times a week | 🔿 |
| e. | four times a week or more | 🔿 |
| **2. If you drink alcohol, how many glasses for you usually drink a day?**  (one glass of alcohol ≈ 0.33l beer, 0.25l wine/ sparkling wine, 0.02l spirits) | | |
| a. | 1-2 glasses per day | 🔿 |
| b. | 3-4 glasses per day | 🔿 |
| c. | 5-6 glasses per day | 🔿 |
| d. | 7-9 glasses per day | 🔿 |
| e. | 10 or more glasses per day | 🔿 |
| **3. How often do you drink six or more glasses of alcohol on one occasion (e.g. at dinner, at a party, etc.)?**  (one glass of alcohol ≈ 0.33l beer, 0.25l wine/ sparkling wine, 0.02l spirits) | | |
| a. | never | 🔿 |
| b. | less than once a month | 🔿 |
| c. | every month | 🔿 |
| d. | every week | 🔿 |
| e. | every day or almost every day | 🔿 |

| **Thank you very much for filling out the questionnaire!** |
| --- |
