# Supplementary material for: A longitudinal analysis on alcohol consumption in patients with cancer undergoing psycho-oncological treatment
Source: Sci Rep. 2025 Jun 20;15:20137. doi: 10.1038/s41598-025-04849-w (PMC12181269; doi:10.1038/s41598-025-04849-w)
Supplement: Supplementary file 2 — Supplementary Material 2 [file 41598_2025_4849_MOESM2_ESM.doc]

**Questionnaire on**

**psychosocial stress**

Patient questionnaire

T1

**A. Personal Data**

| **Today’s date** | | | | Day | | | | | Month | | Year | | **Age** | | ……... | | | | | **Gender** | | | | | | | 🔿 female |
| --- | --- | --- | --- | --- | --- | --- | --- | --- | --- | --- | --- | --- | --- | --- | --- | --- | --- | --- | --- | --- | --- | --- | --- | --- | --- | --- | --- |
|  | | | |  | | |  | |  |  |  |  |  | |  | | | | |  | | | | | | | 🔿 male |
| 1. | **Marital status** | | | |  | | | | | | | | | **Do you live...** | | | | | | |  | | | | | | |
|  | 🔿 | single | | | |  | |  | | | | | | 🔿 | | | in a partnership | | | | | | | | | | |
|  | 🔿 | married | | | |  | |  | | | | | | 🔿 | | | alone | | | | |  |  | | | | |
|  | 🔿 | divorced | | | |  | |  | | | | | | 🔿 | | | other ……………………………. | | | | | | | | | | |
|  | 🔿  🔿 | registered civil partnership  widowed | | | | | | | | | | | |  | | |  | | | | |  |  | | | | |
| 2. | **What is your current work situation?** | | | | | | | | | | | | | | | | | | | | | | | | | | |
|  | 🔿 | employed; full-time | | | | | | | | | | | | | | | | | | | | | | | | | |
|  | 🔿 | employed; part-time | | | | | | | | | | | | | |  | | |  | | | | |  | | | |
|  | 🔿 | unemployed since ________________ | | | | | | | | | | | | | | | | | | | | | | | | | |
|  | 🔿 | retired/retired since ________________  ***If retired:*** 🔿 due to age  🔿 reduced earning capacity pension  🔿 early retirement, but no reduced earning capacity pension | | | | | | | | | | | | | | | | | | | | | | | | | |
|  | 🔿 | housewife/househusband | | | | | | | | | | | | | | | | | | | | | | | | | |
|  | 🔿 | other ………………………………………… | | | | | | | | | | | | | | | | | | | | | | | | | |
| 3. | **Are you currently on sick leave (unable to work)?** | | | | | | | | | | | | | | | | | | | | | | | | | | |
|  | 🔿 | | no | | | | | | | | | | | | | month | | | | year | | | | |  | | |
|  | 🔿 | | yes **since when?** | | | | | | | | | | | | |  | |  | |  | | | |  | |  | |

**B.** Questions about the disease and treatment

| **1.** | **What cancer has been diagnosed?** ………………………………….. | | | | | | | | | | |
| --- | --- | --- | --- | --- | --- | --- | --- | --- | --- | --- | --- |
| 2. | **When was the first diagnosis?** | | | | | | | | | | |
|  |  | | Date of diagnosis: | month | | year | | |  | | |
|  |  | |  |  |  |  |  | |  | | |
| 3. | What treatments have you undergone or are undergoing? | | | | | | | | | | |
| What treatments have you undergone or are undergoing? |  |  | | | | | | **completed** | | **ongoing** | **planned** |
|  | 🔿 | surgery | | | | | | 🔿 | | 🔿 | 🔿 |
|  | 🔿 | radiotherapy | | | | | | 🔿 | | 🔿 | 🔿 |
|  | 🔿 | chemotherapy | | | | | | 🔿 | | 🔿 | 🔿 |
|  | 🔿 | anti-hormonal therapy | | | | | | 🔿 | | 🔿 | 🔿 |
|  | 🔿 | stem cell therapy | | | | | | 🔿 | | 🔿 | 🔿 |
|  | 🔿 | other therapies (antibodies, interferon...) | | | | | | 🔿 | | 🔿 | 🔿 |
|  | 🔿 | pain therapy | | | | | | 🔿 | | 🔿 | 🔿 |
|  | 🔿 | *other therapy ……………………………* | | | | | | 🔿 | | 🔿 | 🔿 |
| 4. | **Do you also have one or more of the diseases listed?** | | | | | | | | | | |
|  | 🔿 | heart disease and circulatory disorders | | | | | | | | | |
|  | 🔿 | diseases of the nervous system | | | | | | | | | |
|  | 🔿 | diseases of the musculoskeletal system and inflammatory diseases | | | | | | | | | |
|  | 🔿 | diseases of the respiratory tract | | | | | | | | | |
|  | 🔿 | stomach disease/diseases of the digestive system | | | | | | | | | |
|  | 🔿 | bladder and kidney diseases | | | | | | | | | |
|  | 🔿 | hormonal diseases (e.g. diabetes) | | | | | | | | | |
|  | 🔿 | skin diseases | | | | | | | | | |
|  | 🔿 | other ………………………………………………………………………………………… | | | | | | | | | |
| 5. | **Are you currently receiving psychological or psychiatric treatment?** | | | | | | | | | | |
|  | yes 🔿 no 🔿 | | | | | | | | | | |
|  | **If yes, who is your treatment provider?** | | | | | | | | | | |
|  | ………………………………………………………………………………………………………... | | | | | | | | | | |

**C. Questions about current stresses**


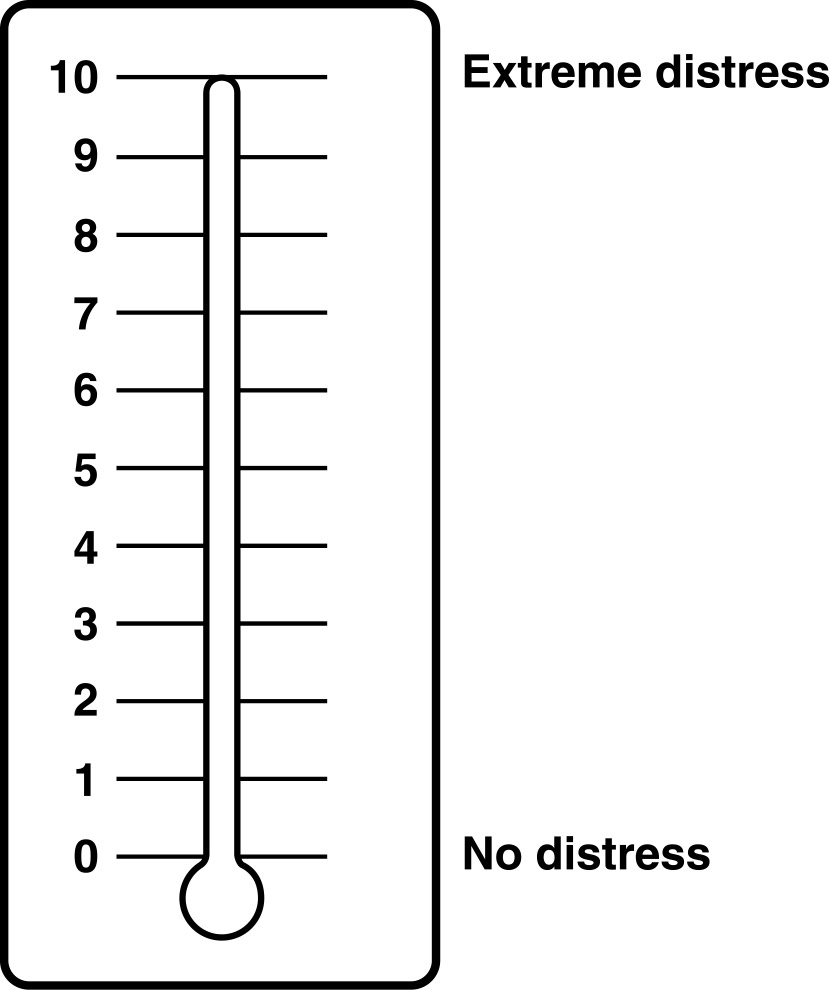


Extremely stressed

No distress

**Instructions:**

**1.** Please circle on the thermometer

the number on the right (0-10) that best describes

best describes how stressed you have felt

**in the last week including today.**

| **2.** Please indicate whether you have had problems in any of the following areas in the last week, including today. Check **YES** or **NO** for each area. | | | | | | |
| --- | --- | --- | --- | --- | --- | --- |
|  | | | | | | |
| **YES** | **NO** |  |  | **YES** | **NO** |  |
|  |  | **Practical problems** |  |  |  | **Physical problems** |
| 🔿 | 🔿 | housing situation |  | 🔿 | 🔿 | pain |
| 🔿 | 🔿 | insurance |  | 🔿 | 🔿 | nausea |
| 🔿 | 🔿 | work/ school |  | 🔿 | 🔿 | exhaustion |
| 🔿 | 🔿 | transportation |  | 🔿 | 🔿 | sleep |
| 🔿 | 🔿 | childcare |  | 🔿 | 🔿 | movement/ mobility |
|  |  |  |  | 🔿 | 🔿 | washing, dressing |
|  |  | **Family problems** |  | 🔿 | 🔿 | appearance |
| 🔿 | 🔿 | dealing with your partner |  | 🔿 | 🔿 | breathing |
| 🔿 | 🔿 | dealing with your children |  | 🔿 | 🔿 | inflammation in the mouth |
|  |  |  |  | 🔿 | 🔿 | eating/ nutrition |
|  |  | **Emotional problems** |  | 🔿 | 🔿 | indigestion |
| 🔿 | 🔿 | worries |  | 🔿 | 🔿 | constipation |
| 🔿 | 🔿 | fears |  | 🔿 | 🔿 | diarrhea |
| 🔿 | 🔿 | sadness |  | 🔿 | 🔿 | changes in urination |
| 🔿 | 🔿 | depression |  | 🔿 | 🔿 | fever |
| 🔿 | 🔿 | nervosity |  | 🔿 | 🔿 | dry/ itchy skin |
| 🔿 | 🔿 | loss of interest in |  | 🔿 | 🔿 | dry/ stuffy nose |
|  |  | everyday activities |  | 🔿 | 🔿 | tingling in hands/ feet |
|  |  |  |  | 🔿 | 🔿 | feeling swollen/ puffy |
|  |  | **Spiritual/ religious matters** |  | 🔿 | 🔿 | memory/ concentration |
| 🔿 | 🔿 | in relation to God |  | 🔿 | 🔿 | sexual problems |
| 🔿 | 🔿 | loss of faith |  |  |  |  |
|  |  |  |  |  |  |  |
| **Other problems**  ………………………………………………………………………………………………………………………. | | | | | | |

| **3. To what extent have you felt affected by the following complaints in the last 2 weeks?** | | | | | |
| --- | --- | --- | --- | --- | --- |
|  |  | **not at all** | **on individual days** | **on more than half of all days** | **almost every day** |
| 1. | Nervousness, anxiety, or tension | 🔿 | 🔿 | 🔿 | 🔿 |
| 2. | Not being able to stop or control worries | 🔿 | 🔿 | 🔿 | 🔿 |
| 3. | Excessive worry about various matters | 🔿 | 🔿 | 🔿 | 🔿 |
| 4. | Difficulty relaxing | 🔿 | 🔿 | 🔿 | 🔿 |
| 5. | Restlessness, making it difficult to sit still | 🔿 | 🔿 | 🔿 | 🔿 |
| 6. | Quick anger or irritability | 🔿 | 🔿 | 🔿 | 🔿 |
| 7. | Feeling anxious, as if something bad is going to happen | 🔿 | 🔿 | 🔿 | 🔿 |

| **4. To what extent have you felt affected by the following complaints in the last 2 weeks?** | | | | | |
| --- | --- | --- | --- | --- | --- |
|  |  | **not at all** | **on individual days** | **on more than half of all days** | **almost every day** |
| 1. | Little interest or pleasure in activities | 🔿 | 🔿 | 🔿 | 🔿 |
| 2. | Depression, melancholy, or hopelessness | 🔿 | 🔿 | 🔿 | 🔿 |
| 3. | Difficulty falling asleep or staying asleep, or increased sleep | 🔿 | 🔿 | 🔿 | 🔿 |
| 4. | Tiredness or feeling of having no energy | 🔿 | 🔿 | 🔿 | 🔿 |
| 5. | Decreased appetite or excessive need to eat | 🔿 | 🔿 | 🔿 | 🔿 |
| 6. | Poor opinion of self; feeling like a failure or having let the family down | 🔿 | 🔿 | 🔿 | 🔿 |
| 7. | Difficulty concentrating on something, e.g. reading the newspaper, or watching TV | 🔿 | 🔿 | 🔿 | 🔿 |
| 8. | Were your movements or speech so slowed down that others would notice? Or, on the contrary, were you "fidgety" or restless and therefore had a stronger urge to move than usual? | 🔿 | 🔿 | 🔿 | 🔿 |
| 9. | Thoughts that you would rather be dead or want to harm yourself | 🔿 | 🔿 | 🔿 | 🔿 |

**D. Questions about alcohol consumption**

| **Some people try to cope with their problems, pain, and/or other stresses by self-medication, e.g. drinking alcohol.**  **Please answer the following questions:** | | |
| --- | --- | --- |
| **1. How often do you drink alcohol?** | | |
| a. | never | 🔿 |
| b. | once a month or less | 🔿 |
| c. | two to four times a month | 🔿 |
| d. | two to three times a week | 🔿 |
| e. | four times a week or more | 🔿 |
| **2. If you drink alcohol, how many glasses for you usually drink a day?**  (one glass of alcohol ≈ 0.33l beer, 0.25l wine/ sparkling wine, 0.02l spirits) | | |
| a. | 1-2 glasses per day | 🔿 |
| b. | 3-4 glasses per day | 🔿 |
| c. | 5-6 glasses per day | 🔿 |
| d. | 7-9 glasses per day | 🔿 |
| e. | 10 or more glasses per day | 🔿 |
| **3. How often do you drink six or more glasses of alcohol on one occasion (e.g. at dinner, at a party, etc.)?**  (one glass of alcohol ≈ 0.33l beer, 0.25l wine/ sparkling wine, 0.02l spirits) | | |
| a. | never | 🔿 |
| b. | less than once a month | 🔿 |
| c. | every month | 🔿 |
| d. | every week | 🔿 |
| e. | every day or almost every day | 🔿 |

| **Thank you very much for filling out the questionnaire!** |
| --- |
